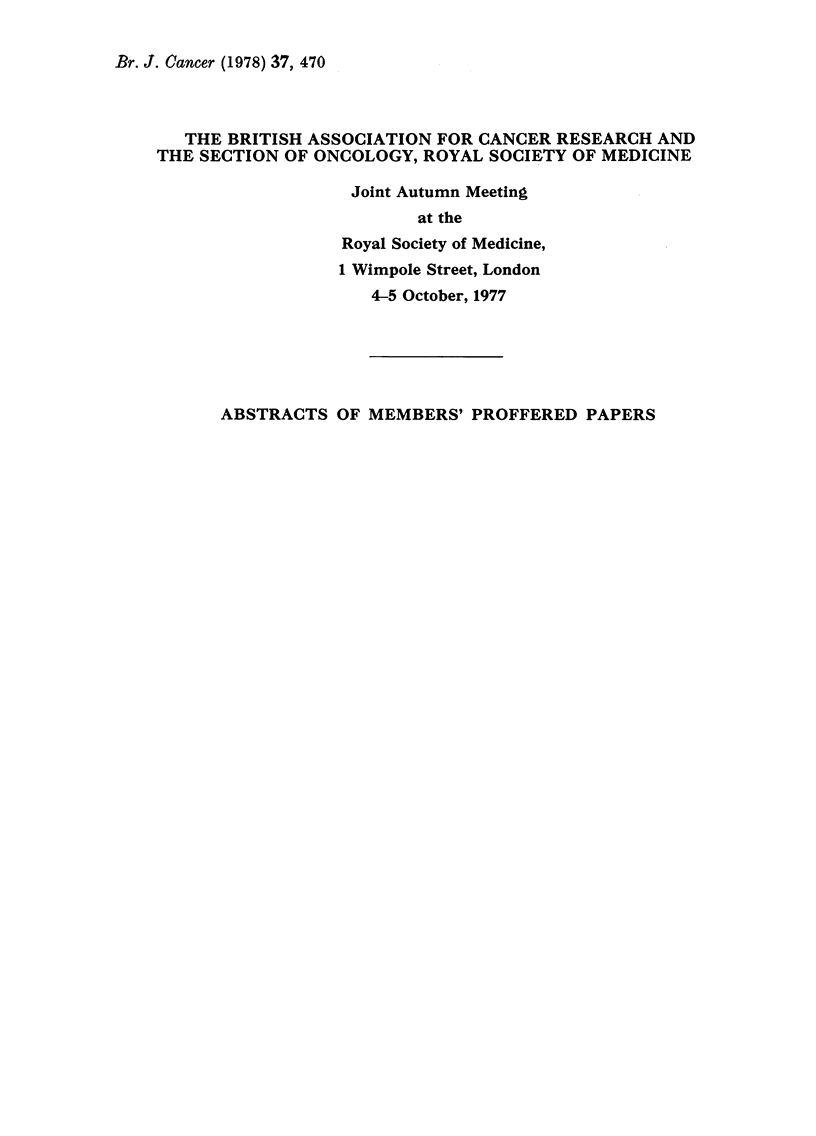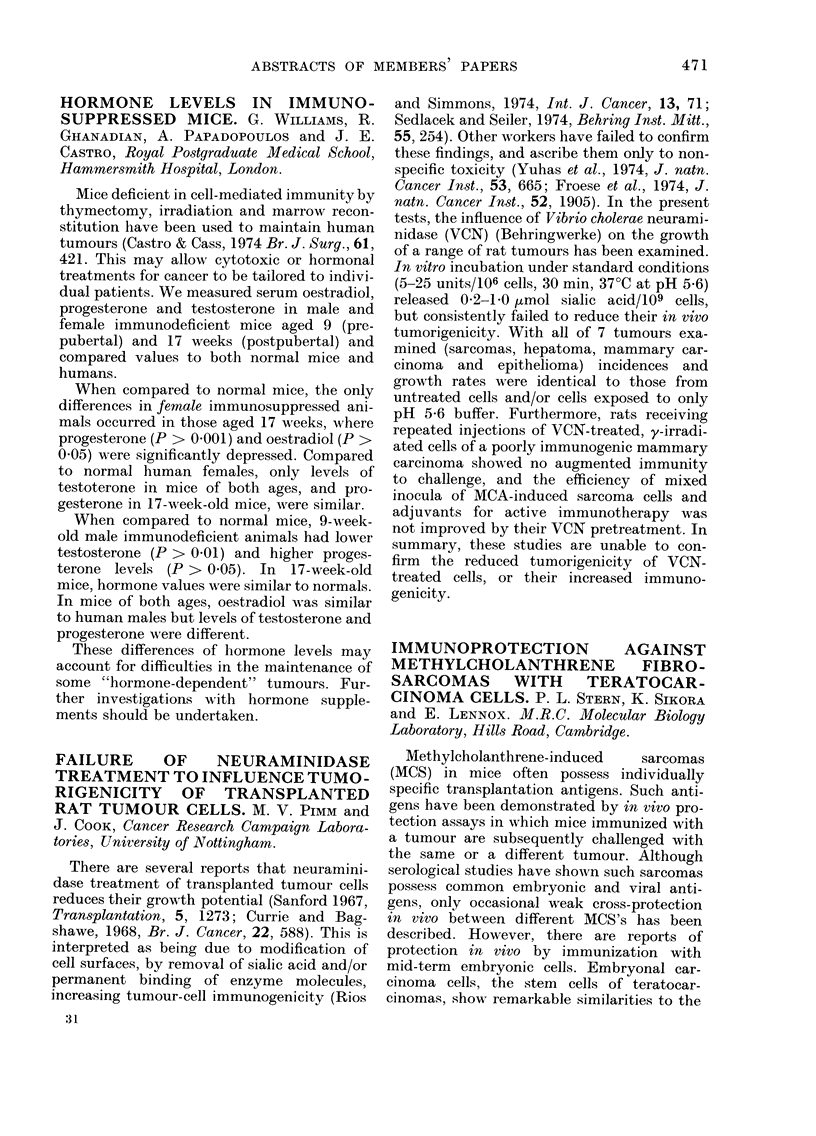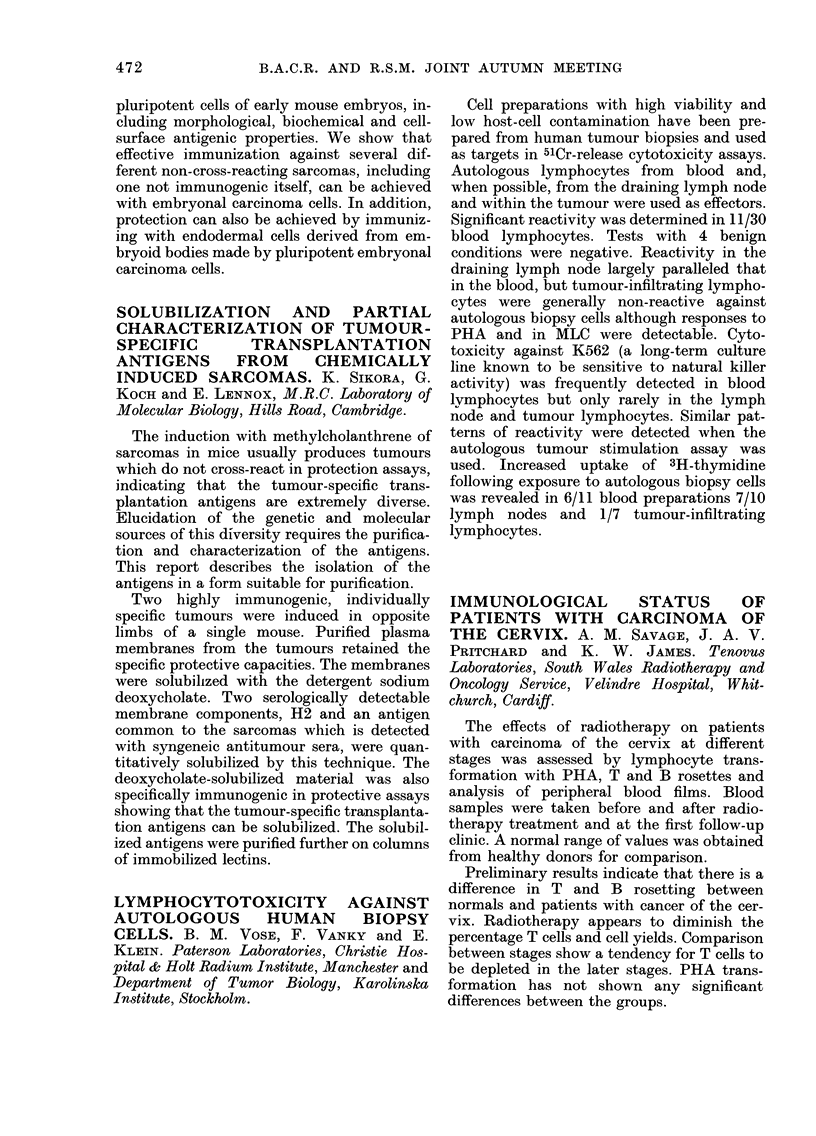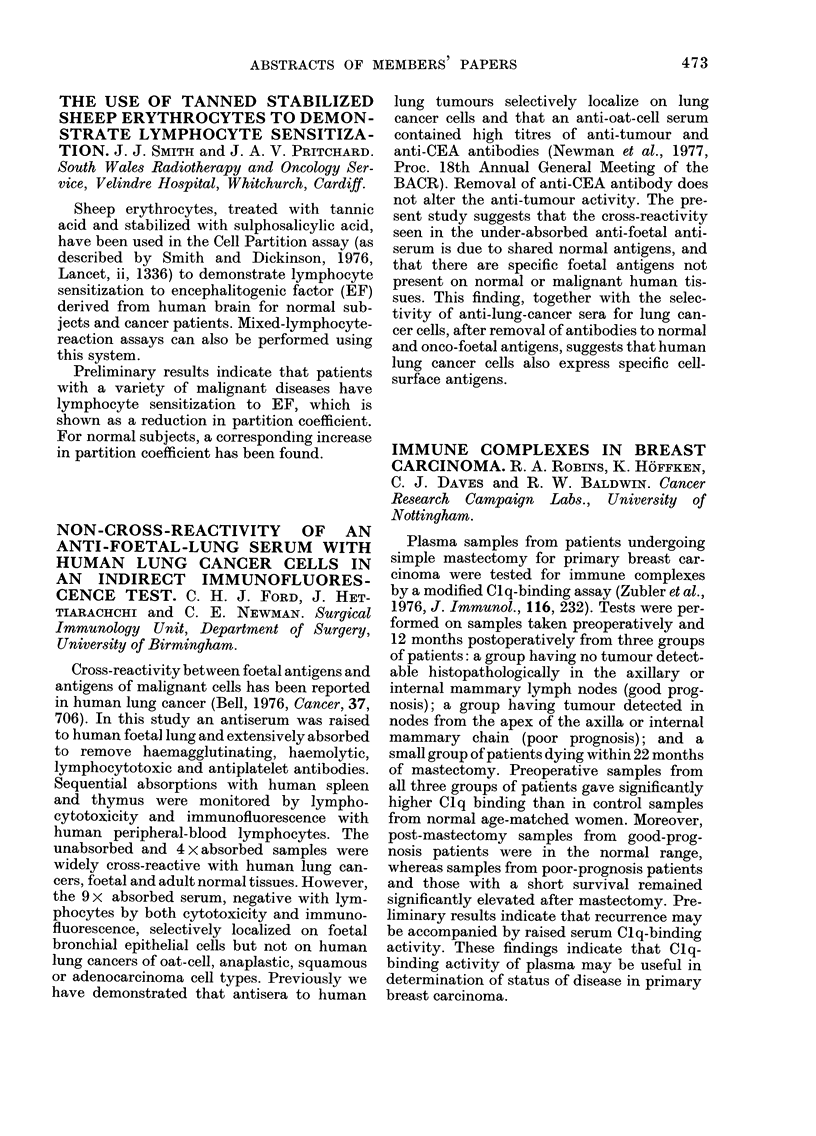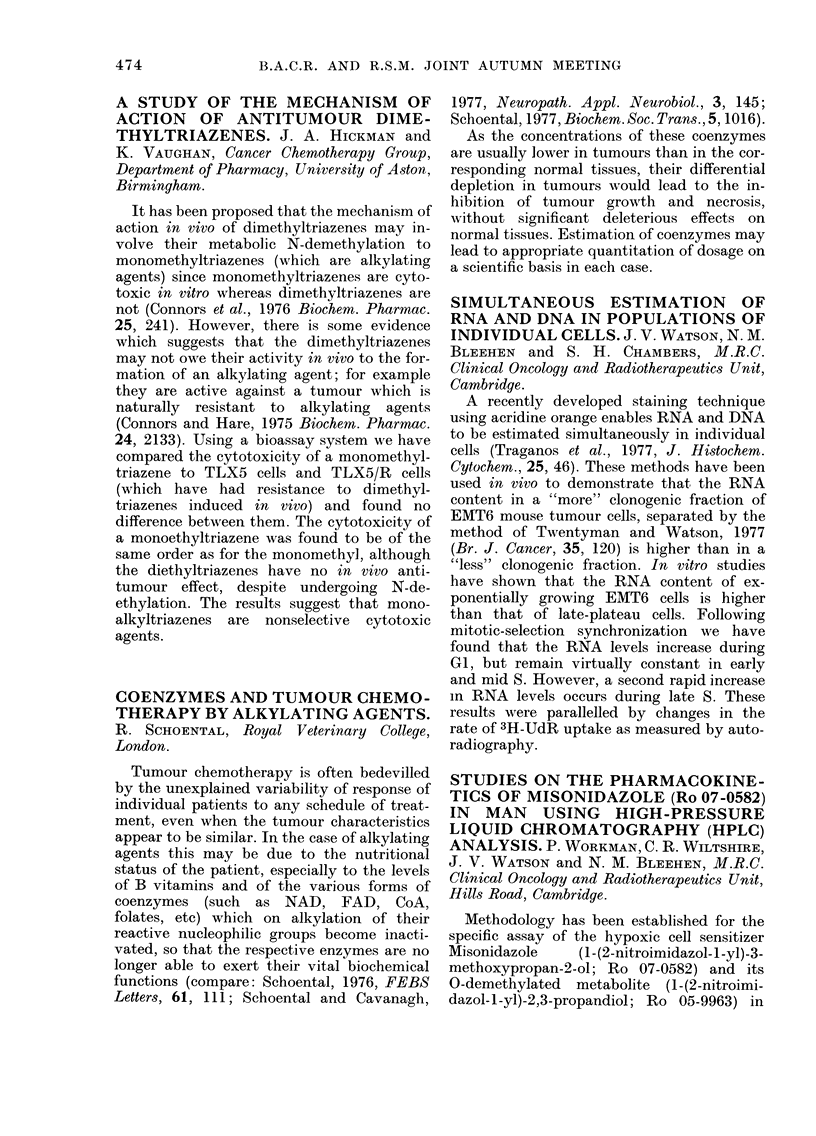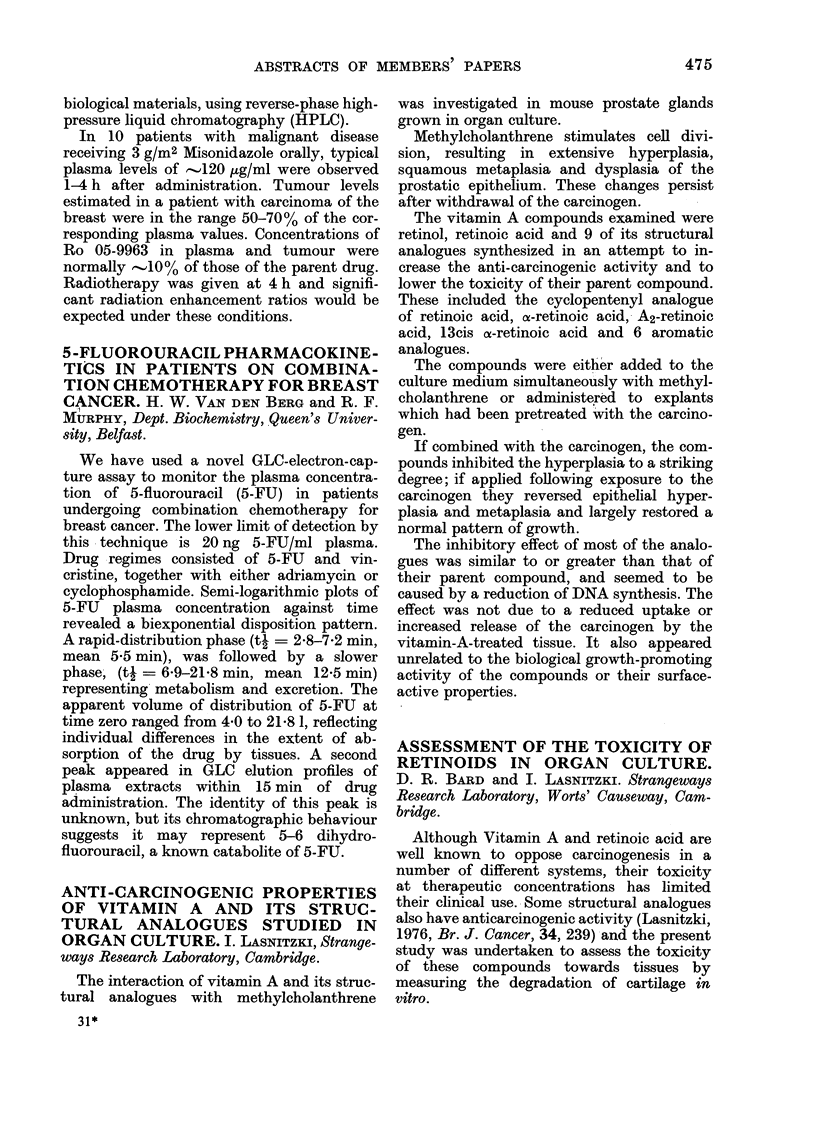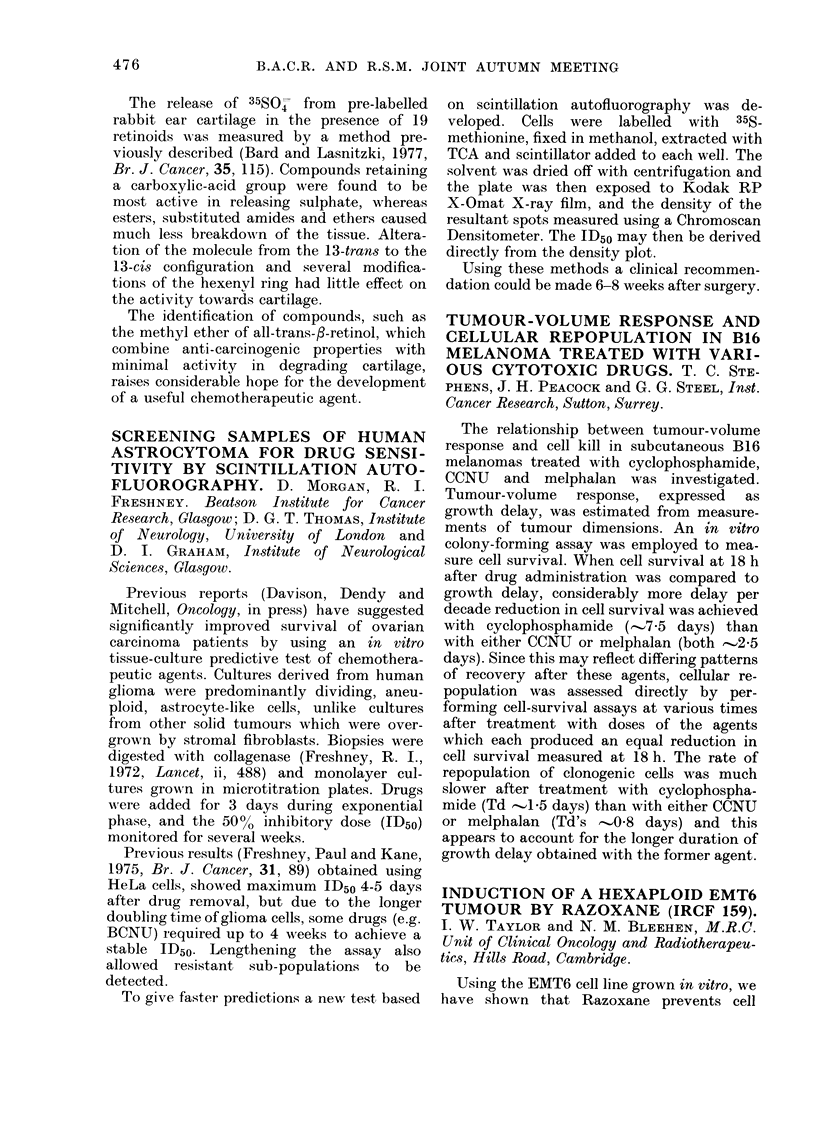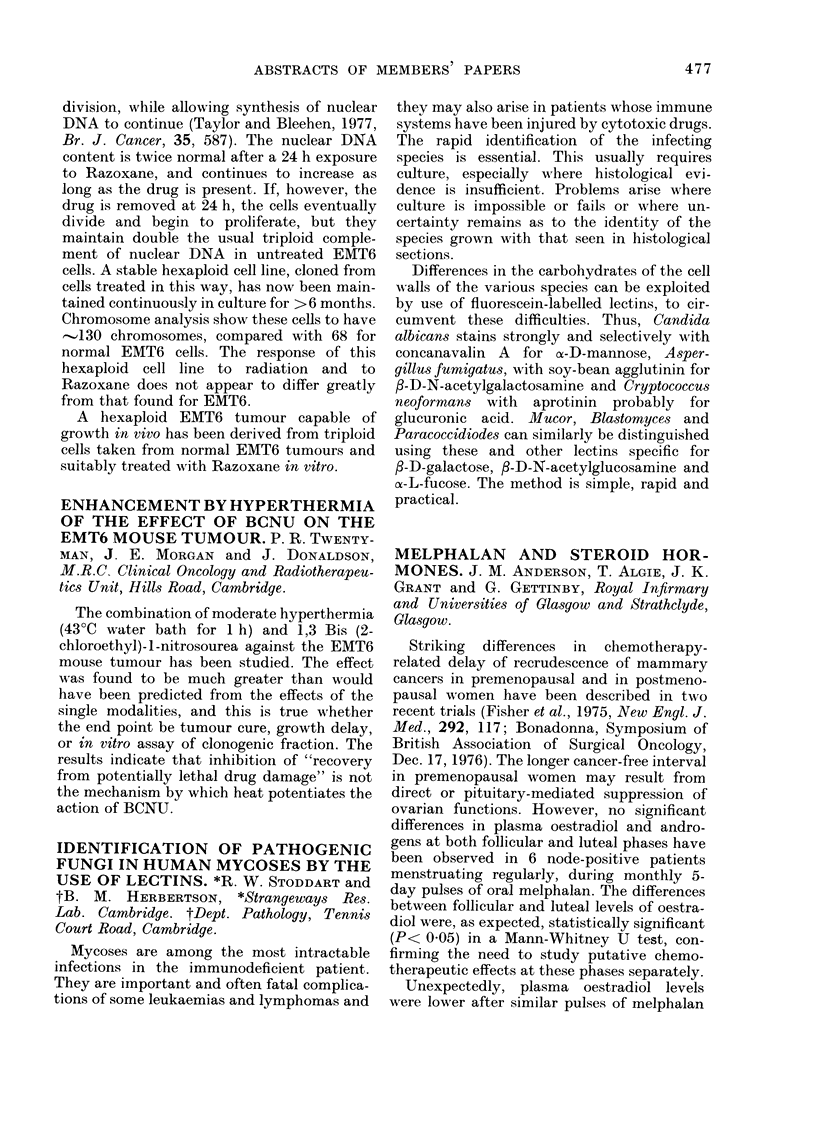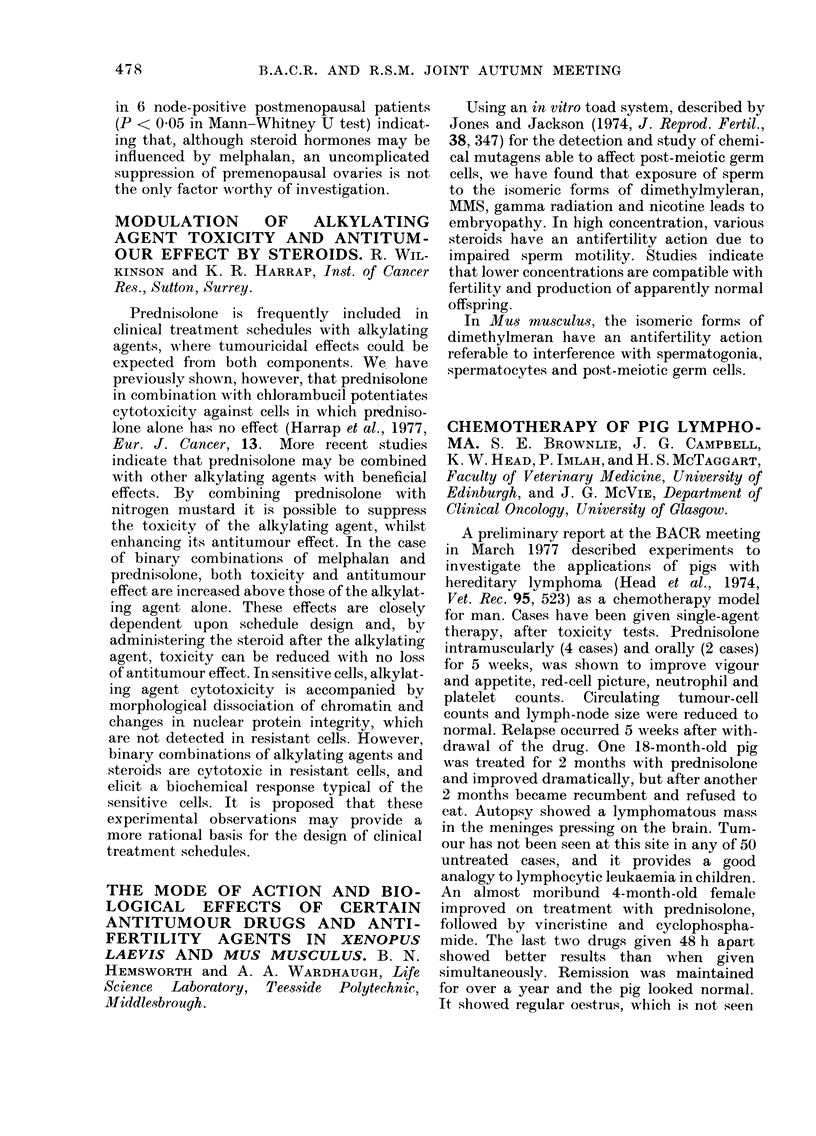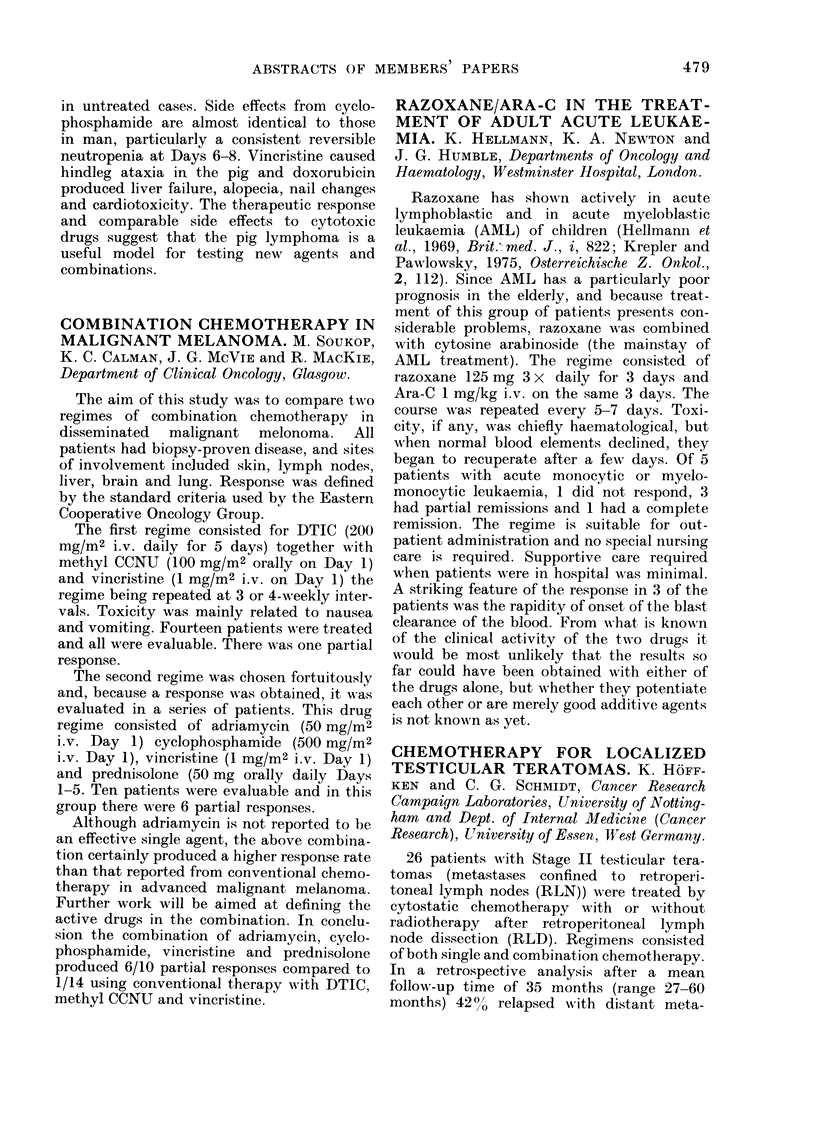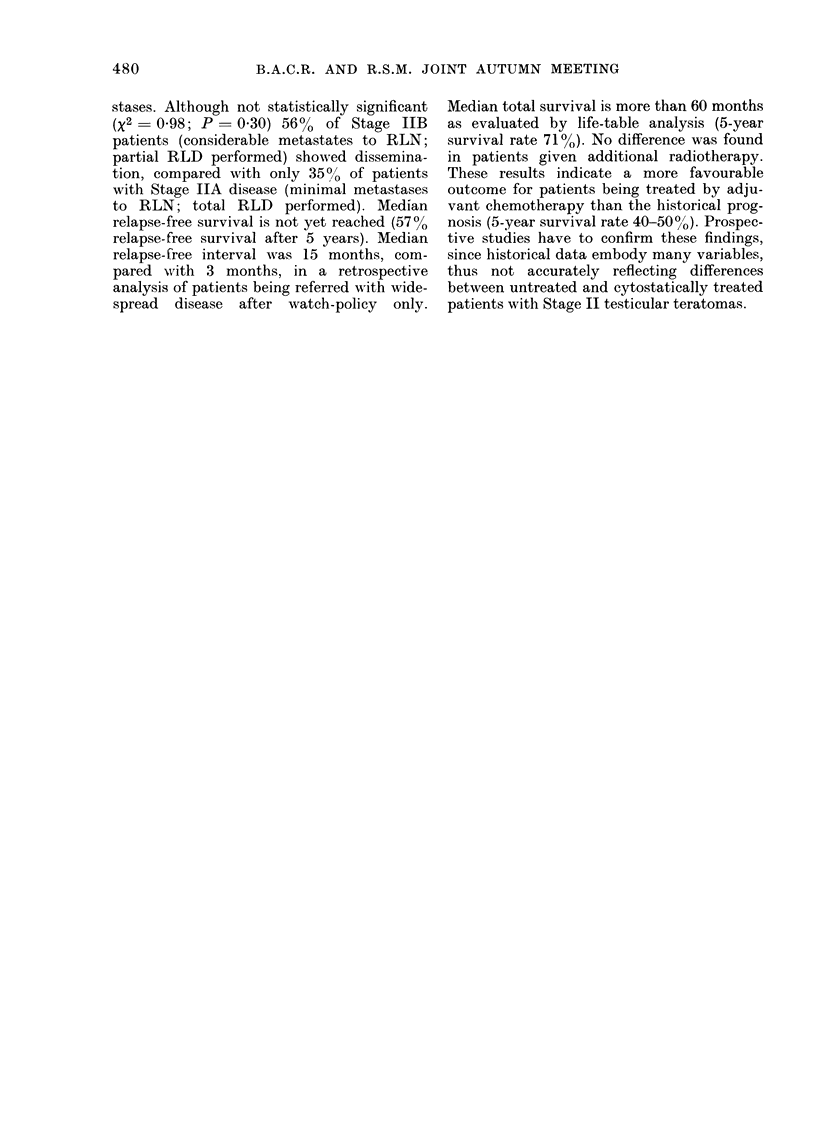# B.A.C.R. and R.S.M. Joint Autumn Meeting (Abstracts)

**Published:** 1978-03

**Authors:** 


*Br. J. Cancer* (1978) **37**, 470

### THE BRITISH ASSOCIATION FOR CANCER RESEARCH AND THE SECTION OF ONCOLOGY, ROYAL SOCIETY OF MEDICINE

**Joint Autumn Meeting**
**at the**
**Royal Society of Medicine,**
**1 Wimpole Street, London**
**4–5 October, 1977**

---

### ABSTRACTS OF MEMBERS' PROFFERED PAPERS

**HORMONE LEVELS IN IMMUNOSUPPRESSED MICE.** G. WILLIAMS, R. GHANADIAN, A. PAPADOPOULOS and J. E. CASTRO, *Royal Postgraduate Medical School, Hammersmith Hospital, London.*

Mice deficient in cell-mediated immunity by thymectomy, irradiation and marrow reconstitution have been used to maintain human tumours (Castro & Cass, 1974 *Br. J. Surg.*, **61**, 421. This may allow cytotoxic or hormonal treatments for cancer to be tailored to individual patients. We measured serum oestradiol, progesterone and testosterone in male and female immunodeficient mice aged 9 (prepubertal) and 17 weeks (postpubertal) and compared values to both normal mice and humans.

When compared to normal mice, the only differences in *female* immunosuppressed animals occurred in those aged 17 weeks, where progesterone ($P > 0{\cdot}001$) and oestradiol ($P > 0{\cdot}05$) were significantly depressed. Compared to normal human females, only levels of testoterone in mice of both ages, and progesterone in 17-week-old mice, were similar.

When compared to normal mice, 9-week-old male immunodeficient animals had lower testosterone ($P > 0{\cdot}01$) and higher progesterone levels ($P > 0{\cdot}05$). In 17-week-old mice, hormone values were similar to normals. In mice of both ages, oestradiol was similar to human males but levels of testosterone and progesterone were different.

These differences of hormone levels may account for difficulties in the maintenance of some "hormone-dependent" tumours. Further investigations with hormone supplements should be undertaken.

**FAILURE OF NEURAMINIDASE TREATMENT TO INFLUENCE TUMORIGENICITY OF TRANSPLANTED RAT TUMOUR CELLS.** M. V. PIMM and J. COOK, *Cancer Research Campaign Laboratories, University of Nottingham.*

There are several reports that neuraminidase treatment of transplanted tumour cells reduces their growth potential (Sanford 1967, *Transplantation*, **5**, 1273; Currie and Bagshawe, 1968, *Br. J. Cancer*, **22**, 588). This is interpreted as being due to modification of cell surfaces, by removal of sialic acid and/or permanent binding of enzyme molecules, increasing tumour-cell immunogenicity (Rios and Simmons, 1974, *Int. J. Cancer*, **13**, 71; Sedlacek and Seiler, 1974, *Behring Inst. Mitt.*, **55**, 254). Other workers have failed to confirm these findings, and ascribe them only to non-specific toxicity (Yuhas *et al.*, 1974, *J. natn. Cancer Inst.*, **53**, 665; Froese *et al.*, 1974, *J. natn. Cancer Inst.*, **52**, 1905). In the present tests, the influence of *Vibrio cholerae* neuraminidase (VCN) (Behringwerke) on the growth of a range of rat tumours has been examined. *In vitro* incubation under standard conditions (5–25 units/$10^6$ cells, 30 min, 37°C at pH 5·6) released 0·2–1·0 $\mu$mol sialic acid/$10^9$ cells, but consistently failed to reduce their *in vivo* tumorigenicity. With all of 7 tumours examined (sarcomas, hepatoma, mammary carcinoma and epithelioma) incidences and growth rates were identical to those from untreated cells and/or cells exposed to only pH 5·6 buffer. Furthermore, rats receiving repeated injections of VCN-treated, $\gamma$-irradiated cells of a poorly immunogenic mammary carcinoma showed no augmented immunity to challenge, and the efficiency of mixed inocula of MCA-induced sarcoma cells and adjuvants for active immunotherapy was not improved by their VCN pretreatment. In summary, these studies are unable to confirm the reduced tumorigenicity of VCN-treated cells, or their increased immunogenicity.

**IMMUNOPROTECTION AGAINST METHYLCHOLANTHRENE FIBROSARCOMAS WITH TERATOCARCINOMA CELLS.** P. L. STERN, K. SIKORA and E. LENNOX. *M.R.C. Molecular Biology Laboratory, Hills Road, Cambridge.*

Methylcholanthrene-induced sarcomas (MCS) in mice often possess individually specific transplantation antigens. Such antigens have been demonstrated by *in vivo* protection assays in which mice immunized with a tumour are subsequently challenged with the same or a different tumour. Although serological studies have shown such sarcomas possess common embryonic and viral antigens, only occasional weak cross-protection *in vivo* between different MCS's has been described. However, there are reports of protection *in vivo* by immunization with mid-term embryonic cells. Embryonal carcinoma cells, the stem cells of teratocarcinomas, show remarkable similarities to the pluripotent cells of early mouse embryos, including morphological, biochemical and cell-surface antigenic properties. We show that effective immunization against several different non-cross-reacting sarcomas, including one not immunogenic itself, can be achieved with embryonal carcinoma cells. In addition, protection can also be achieved by immunizing with endodermal cells derived from embryoid bodies made by pluripotent embryonal carcinoma cells.

### SOLUBILIZATION AND PARTIAL CHARACTERIZATION OF TUMOUR-SPECIFIC TRANSPLANTATION ANTIGENS FROM CHEMICALLY INDUCED SARCOMAS.

K. SIKORA, G. KOCH and E. LENNOX, *M.R.C. Laboratory of Molecular Biology, Hills Road, Cambridge.*

The induction with methylcholanthrene of sarcomas in mice usually produces tumours which do not cross-react in protection assays, indicating that the tumour-specific transplantation antigens are extremely diverse. Elucidation of the genetic and molecular sources of this diversity requires the purification and characterization of the antigens. This report describes the isolation of the antigens in a form suitable for purification.

Two highly immunogenic, individually specific tumours were induced in opposite limbs of a single mouse. Purified plasma membranes from the tumours retained the specific protective capacities. The membranes were solubilized with the detergent sodium deoxycholate. Two serologically detectable membrane components, H2 and an antigen common to the sarcomas which is detected with syngeneic antitumour sera, were quantitatively solubilized by this technique. The deoxycholate-solubilized material was also specifically immunogenic in protective assays showing that the tumour-specific transplantation antigens can be solubilized. The solubilized antigens were purified further on columns of immobilized lectins.

### LYMPHOCYTOTOXICITY AGAINST AUTOLOGOUS HUMAN BIOPSY CELLS.

B. M. VOSE, F. VANKY and E. KLEIN. *Paterson Laboratories, Christie Hospital & Holt Radium Institute, Manchester* and *Department of Tumor Biology, Karolinska Institute, Stockholm.*

Cell preparations with high viability and low host-cell contamination have been prepared from human tumour biopsies and used as targets in $^{51}$Cr-release cytotoxicity assays. Autologous lymphocytes from blood and, when possible, from the draining lymph node and within the tumour were used as effectors. Significant reactivity was determined in 11/30 blood lymphocytes. Tests with 4 benign conditions were negative. Reactivity in the draining lymph node largely paralleled that in the blood, but tumour-infiltrating lymphocytes were generally non-reactive against autologous biopsy cells although responses to PHA and in MLC were detectable. Cytotoxicity against K562 (a long-term culture line known to be sensitive to natural killer activity) was frequently detected in blood lymphocytes but only rarely in the lymph node and tumour lymphocytes. Similar patterns of reactivity were detected when the autologous tumour stimulation assay was used. Increased uptake of $^{3}$H-thymidine following exposure to autologous biopsy cells was revealed in 6/11 blood preparations 7/10 lymph nodes and 1/7 tumour-infiltrating lymphocytes.

### IMMUNOLOGICAL STATUS OF PATIENTS WITH CARCINOMA OF THE CERVIX.

A. M. SAVAGE, J. A. V. PRITCHARD and K. W. JAMES. *Tenovus Laboratories, South Wales Radiotherapy and Oncology Service, Velindre Hospital, Whitchurch, Cardiff.*

The effects of radiotherapy on patients with carcinoma of the cervix at different stages was assessed by lymphocyte transformation with PHA, T and B rosettes and analysis of peripheral blood films. Blood samples were taken before and after radiotherapy treatment and at the first follow-up clinic. A normal range of values was obtained from healthy donors for comparison.

Preliminary results indicate that there is a difference in T and B rosetting between normals and patients with cancer of the cervix. Radiotherapy appears to diminish the percentage T cells and cell yields. Comparison between stages show a tendency for T cells to be depleted in the later stages. PHA transformation has not shown any significant differences between the groups.

**THE USE OF TANNED STABILIZED SHEEP ERYTHROCYTES TO DEMONSTRATE LYMPHOCYTE SENSITIZATION.** J. J. SMITH and J. A. V. PRITCHARD. *South Wales Radiotherapy and Oncology Service, Velindre Hospital, Whitchurch, Cardiff.*

Sheep erythrocytes, treated with tannic acid and stabilized with sulphosalicylic acid, have been used in the Cell Partition assay (as described by Smith and Dickinson, 1976, Lancet, ii, 1336) to demonstrate lymphocyte sensitization to encephalitogenic factor (EF) derived from human brain for normal subjects and cancer patients. Mixed-lymphocyte-reaction assays can also be performed using this system.

Preliminary results indicate that patients with a variety of malignant diseases have lymphocyte sensitization to EF, which is shown as a reduction in partition coefficient. For normal subjects, a corresponding increase in partition coefficient has been found.

**NON-CROSS-REACTIVITY OF AN ANTI-FOETAL-LUNG SERUM WITH HUMAN LUNG CANCER CELLS IN AN INDIRECT IMMUNOFLUORESCENCE TEST.** C. H. J. FORD, J. HETTIARACHCHI and C. E. NEWMAN. *Surgical Immunology Unit, Department of Surgery, University of Birmingham.*

Cross-reactivity between foetal antigens and antigens of malignant cells has been reported in human lung cancer (Bell, 1976, *Cancer*, **37**, 706). In this study an antiserum was raised to human foetal lung and extensively absorbed to remove haemagglutinating, haemolytic, lymphocytotoxic and antiplatelet antibodies. Sequential absorptions with human spleen and thymus were monitored by lymphocytotoxicity and immunofluorescence with human peripheral-blood lymphocytes. The unabsorbed and 4× absorbed samples were widely cross-reactive with human lung cancers, foetal and adult normal tissues. However, the 9× absorbed serum, negative with lymphocytes by both cytotoxicity and immunofluorescence, selectively localized on foetal bronchial epithelial cells but not on human lung cancers of oat-cell, anaplastic, squamous or adenocarcinoma cell types. Previously we have demonstrated that antisera to human lung tumours selectively localize on lung cancer cells and that an anti-oat-cell serum contained high titres of anti-tumour and anti-CEA antibodies (Newman *et al.*, 1977, Proc. 18th Annual General Meeting of the BACR). Removal of anti-CEA antibody does not alter the anti-tumour activity. The present study suggests that the cross-reactivity seen in the under-absorbed anti-foetal antiserum is due to shared normal antigens, and that there are specific foetal antigens not present on normal or malignant human tissues. This finding, together with the selectivity of anti-lung-cancer sera for lung cancer cells, after removal of antibodies to normal and onco-foetal antigens, suggests that human lung cancer cells also express specific cell-surface antigens.

**IMMUNE COMPLEXES IN BREAST CARCINOMA.** R. A. ROBINS, K. HÖFFKEN, C. J. DAVES and R. W. BALDWIN. *Cancer Research Campaign Labs., University of Nottingham.*

Plasma samples from patients undergoing simple mastectomy for primary breast carcinoma were tested for immune complexes by a modified C1q-binding assay (Zubler *et al.*, 1976, *J. Immunol.*, **116**, 232). Tests were performed on samples taken preoperatively and 12 months postoperatively from three groups of patients: a group having no tumour detectable histopathologically in the axillary or internal mammary lymph nodes (good prognosis); a group having tumour detected in nodes from the apex of the axilla or internal mammary chain (poor prognosis); and a small group of patients dying within 22 months of mastectomy. Preoperative samples from all three groups of patients gave significantly higher C1q binding than in control samples from normal age-matched women. Moreover, post-mastectomy samples from good-prognosis patients were in the normal range, whereas samples from poor-prognosis patients and those with a short survival remained significantly elevated after mastectomy. Preliminary results indicate that recurrence may be accompanied by raised serum C1q-binding activity. These findings indicate that C1q-binding activity of plasma may be useful in determination of status of disease in primary breast carcinoma.

**A STUDY OF THE MECHANISM OF ACTION OF ANTITUMOUR DIMETHYLTRIAZENES.** J. A. HICKMAN and K. VAUGHAN, *Cancer Chemotherapy Group, Department of Pharmacy, University of Aston, Birmingham.*

It has been proposed that the mechanism of action *in vivo* of dimethyltriazenes may involve their metabolic N-demethylation to monomethyltriazenes (which are alkylating agents) since monomethyltriazenes are cytotoxic *in vitro* whereas dimethyltriazenes are not (Connors *et al.*, 1976 *Biochem. Pharmac.* **25**, 241). However, there is some evidence which suggests that the dimethyltriazenes may not owe their activity *in vivo* to the formation of an alkylating agent; for example they are active against a tumour which is naturally resistant to alkylating agents (Connors and Hare, 1975 *Biochem. Pharmac.* **24**, 2133). Using a bioassay system we have compared the cytotoxicity of a monomethyltriazene to TLX5 cells and TLX5/R cells (which have had resistance to dimethyltriazenes induced *in vivo*) and found no difference between them. The cytotoxicity of a monoethyltriazene was found to be of the same order as for the monomethyl, although the diethyltriazenes have no *in vivo* antitumour effect, despite undergoing N-deethylation. The results suggest that monoalkyltriazenes are nonselective cytotoxic agents.

**COENZYMES AND TUMOUR CHEMOTHERAPY BY ALKYLATING AGENTS.** R. SCHOENTAL, *Royal Veterinary College, London.*

Tumour chemotherapy is often bedevilled by the unexplained variability of response of individual patients to any schedule of treatment, even when the tumour characteristics appear to be similar. In the case of alkylating agents this may be due to the nutritional status of the patient, especially to the levels of B vitamins and of the various forms of coenzymes (such as NAD, FAD, CoA, folates, etc) which on alkylation of their reactive nucleophilic groups become inactivated, so that the respective enzymes are no longer able to exert their vital biochemical functions (compare: Schoental, 1976, *FEBS Letters*, **61**, 111; Schoental and Cavanagh, 1977, *Neuropath. Appl. Neurobiol.*, **3**, 145; Schoental, 1977, *Biochem. Soc. Trans.*, **5**, 1016).

As the concentrations of these coenzymes are usually lower in tumours than in the corresponding normal tissues, their differential depletion in tumours would lead to the inhibition of tumour growth and necrosis, without significant deleterious effects on normal tissues. Estimation of coenzymes may lead to appropriate quantitation of dosage on a scientific basis in each case.

**SIMULTANEOUS ESTIMATION OF RNA AND DNA IN POPULATIONS OF INDIVIDUAL CELLS.** J. V. WATSON, N. M. BLEEHEN and S. H. CHAMBERS, *M.R.C. Clinical Oncology and Radiotherapeutics Unit, Cambridge.*

A recently developed staining technique using acridine orange enables RNA and DNA to be estimated simultaneously in individual cells (Traganos *et al.*, 1977, *J. Histochem. Cytochem.*, **25**, 46). These methods have been used *in vivo* to demonstrate that the RNA content in a "more" clonogenic fraction of EMT6 mouse tumour cells, separated by the method of Twentyman and Watson, 1977 (*Br. J. Cancer*, **35**, 120) is higher than in a "less" clonogenic fraction. *In vitro* studies have shown that the RNA content of exponentially growing EMT6 cells is higher than that of late-plateau cells. Following mitotic-selection synchronization we have found that the RNA levels increase during G1, but remain virtually constant in early and mid S. However, a second rapid increase in RNA levels occurs during late S. These results were parallelled by changes in the rate of $^{3}$H-UdR uptake as measured by autoradiography.

**STUDIES ON THE PHARMACOKINETICS OF MISONIDAZOLE (Ro 07-0582) IN MAN USING HIGH-PRESSURE LIQUID CHROMATOGRAPHY (HPLC) ANALYSIS.** P. WORKMAN, C. R. WILTSHIRE, J. V. WATSON and N. M. BLEEHEN, *M.R.C. Clinical Oncology and Radiotherapeutics Unit, Hills Road, Cambridge.*

Methodology has been established for the specific assay of the hypoxic cell sensitizer Misonidazole (1-(2-nitroimidazol-1-yl)-3-methoxypropan-2-ol; Ro 07-0582) and its O-demethylated metabolite (1-(2-nitroimidazol-1-yl)-2,3-propandiol; Ro 05-9963) in biological materials, using reverse-phase high-pressure liquid chromatography (HPLC).

In 10 patients with malignant disease receiving 3 g/m$^2$ Misonidazole orally, typical plasma levels of ~120 $\mu$g/ml were observed 1–4 h after administration. Tumour levels estimated in a patient with carcinoma of the breast were in the range 50–70% of the corresponding plasma values. Concentrations of Ro 05-9963 in plasma and tumour were normally ~10% of those of the parent drug. Radiotherapy was given at 4 h and significant radiation enhancement ratios would be expected under these conditions.

### 5-FLUOROURACIL PHARMACOKINETICS IN PATIENTS ON COMBINATION CHEMOTHERAPY FOR BREAST CANCER.

H. W. VAN DEN BERG and R. F. MURPHY, *Dept. Biochemistry, Queen's University, Belfast.*

We have used a novel GLC-electron-capture assay to monitor the plasma concentration of 5-fluorouracil (5-FU) in patients undergoing combination chemotherapy for breast cancer. The lower limit of detection by this technique is 20 ng 5-FU/ml plasma. Drug regimes consisted of 5-FU and vincristine, together with either adriamycin or cyclophosphamide. Semi-logarithmic plots of 5-FU plasma concentration against time revealed a biexponential disposition pattern. A rapid-distribution phase ($t\frac{1}{2}$ = 2·8–7·2 min, mean 5·5 min), was followed by a slower phase, ($t\frac{1}{2}$ = 6·9–21·8 min, mean 12·5 min) representing metabolism and excretion. The apparent volume of distribution of 5-FU at time zero ranged from 4·0 to 21·8 l, reflecting individual differences in the extent of absorption of the drug by tissues. A second peak appeared in GLC elution profiles of plasma extracts within 15 min of drug administration. The identity of this peak is unknown, but its chromatographic behaviour suggests it may represent 5–6 dihydrofluorouracil, a known catabolite of 5-FU.

### ANTI-CARCINOGENIC PROPERTIES OF VITAMIN A AND ITS STRUCTURAL ANALOGUES STUDIED IN ORGAN CULTURE.

I. LASNITZKI, *Strangeways Research Laboratory, Cambridge.*

The interaction of vitamin A and its structural analogues with methylcholanthrene was investigated in mouse prostate glands grown in organ culture.

Methylcholanthrene stimulates cell division, resulting in extensive hyperplasia, squamous metaplasia and dysplasia of the prostatic epithelium. These changes persist after withdrawal of the carcinogen.

The vitamin A compounds examined were retinol, retinoic acid and 9 of its structural analogues synthesized in an attempt to increase the anti-carcinogenic activity and to lower the toxicity of their parent compound. These included the cyclopentenyl analogue of retinoic acid, $\alpha$-retinoic acid, $A_2$-retinoic acid, 13cis $\alpha$-retinoic acid and 6 aromatic analogues.

The compounds were either added to the culture medium simultaneously with methylcholanthrene or administered to explants which had been pretreated with the carcinogen.

If combined with the carcinogen, the compounds inhibited the hyperplasia to a striking degree; if applied following exposure to the carcinogen they reversed epithelial hyperplasia and metaplasia and largely restored a normal pattern of growth.

The inhibitory effect of most of the analogues was similar to or greater than that of their parent compound, and seemed to be caused by a reduction of DNA synthesis. The effect was not due to a reduced uptake or increased release of the carcinogen by the vitamin-A-treated tissue. It also appeared unrelated to the biological growth-promoting activity of the compounds or their surface-active properties.

### ASSESSMENT OF THE TOXICITY OF RETINOIDS IN ORGAN CULTURE.

D. R. BARD and I. LASNITZKI. *Strangeways Research Laboratory, Worts' Causeway, Cambridge.*

Although Vitamin A and retinoic acid are well known to oppose carcinogenesis in a number of different systems, their toxicity at therapeutic concentrations has limited their clinical use. Some structural analogues also have anticarcinogenic activity (Lasnitzki, 1976, *Br. J. Cancer*, **34**, 239) and the present study was undertaken to assess the toxicity of these compounds towards tissues by measuring the degradation of cartilage *in vitro*.

The release of $^{35}SO_4^=$ from pre-labelled rabbit ear cartilage in the presence of 19 retinoids was measured by a method previously described (Bard and Lasnitzki, 1977, *Br. J. Cancer*, **35**, 115). Compounds retaining a carboxylic-acid group were found to be most active in releasing sulphate, whereas esters, substituted amides and ethers caused much less breakdown of the tissue. Alteration of the molecule from the 13-*trans* to the 13-*cis* configuration and several modifications of the hexenyl ring had little effect on the activity towards cartilage.

The identification of compounds, such as the methyl ether of all-trans-$\beta$-retinol, which combine anti-carcinogenic properties with minimal activity in degrading cartilage, raises considerable hope for the development of a useful chemotherapeutic agent.

### SCREENING SAMPLES OF HUMAN ASTROCYTOMA FOR DRUG SENSITIVITY BY SCINTILLATION AUTOFLUOROGRAPHY.

D. MORGAN, R. I. FRESHNEY. *Beatson Institute for Cancer Research, Glasgow*; D. G. T. THOMAS, *Institute of Neurology, University of London* and D. I. GRAHAM, *Institute of Neurological Sciences, Glasgow.*

Previous reports (Davison, Dendy and Mitchell, *Oncology*, in press) have suggested significantly improved survival of ovarian carcinoma patients by using an *in vitro* tissue-culture predictive test of chemotherapeutic agents. Cultures derived from human glioma were predominantly dividing, aneuploid, astrocyte-like cells, unlike cultures from other solid tumours which were overgrown by stromal fibroblasts. Biopsies were digested with collagenase (Freshney, R. I., 1972, *Lancet*, ii, 488) and monolayer cultures grown in microtitration plates. Drugs were added for 3 days during exponential phase, and the 50% inhibitory dose ($ID_{50}$) monitored for several weeks.

Previous results (Freshney, Paul and Kane, 1975, *Br. J. Cancer*, **31**, 89) obtained using HeLa cells, showed maximum $ID_{50}$ 4-5 days after drug removal, but due to the longer doubling time of glioma cells, some drugs (e.g. BCNU) required up to 4 weeks to achieve a stable $ID_{50}$. Lengthening the assay also allowed resistant sub-populations to be detected.

To give faster predictions a new test based on scintillation autofluorography was developed. Cells were labelled with $^{35}S$-methionine, fixed in methanol, extracted with TCA and scintillator added to each well. The solvent was dried off with centrifugation and the plate was then exposed to Kodak RP X-Omat X-ray film, and the density of the resultant spots measured using a Chromoscan Densitometer. The $ID_{50}$ may then be derived directly from the density plot.

Using these methods a clinical recommendation could be made 6–8 weeks after surgery.

### TUMOUR-VOLUME RESPONSE AND CELLULAR REPOPULATION IN B16 MELANOMA TREATED WITH VARIOUS CYTOTOXIC DRUGS.

T. C. STEPHENS, J. H. PEACOCK and G. G. STEEL, *Inst. Cancer Research, Sutton, Surrey.*

The relationship between tumour-volume response and cell kill in subcutaneous B16 melanomas treated with cyclophosphamide, CCNU and melphalan was investigated. Tumour-volume response, expressed as growth delay, was estimated from measurements of tumour dimensions. An *in vitro* colony-forming assay was employed to measure cell survival. When cell survival at 18 h after drug administration was compared to growth delay, considerably more delay per decade reduction in cell survival was achieved with cyclophosphamide ($\sim$7·5 days) than with either CCNU or melphalan (both $\sim$2·5 days). Since this may reflect differing patterns of recovery after these agents, cellular repopulation was assessed directly by performing cell-survival assays at various times after treatment with doses of the agents which each produced an equal reduction in cell survival measured at 18 h. The rate of repopulation of clonogenic cells was much slower after treatment with cyclophosphamide (Td $\sim$1·5 days) than with either CCNU or melphalan (Td's $\sim$0·8 days) and this appears to account for the longer duration of growth delay obtained with the former agent.

### INDUCTION OF A HEXAPLOID EMT6 TUMOUR BY RAZOXANE (IRCF 159).

I. W. TAYLOR and N. M. BLEEHEN, *M.R.C. Unit of Clinical Oncology and Radiotherapeutics, Hills Road, Cambridge.*

Using the EMT6 cell line grown *in vitro*, we have shown that Razoxane prevents cell division, while allowing synthesis of nuclear DNA to continue (Taylor and Bleehen, 1977, *Br. J. Cancer*, **35**, 587). The nuclear DNA content is twice normal after a 24 h exposure to Razoxane, and continues to increase as long as the drug is present. If, however, the drug is removed at 24 h, the cells eventually divide and begin to proliferate, but they maintain double the usual triploid complement of nuclear DNA in untreated EMT6 cells. A stable hexaploid cell line, cloned from cells treated in this way, has now been maintained continuously in culture for $>6$ months. Chromosome analysis show these cells to have $\sim$130 chromosomes, compared with 68 for normal EMT6 cells. The response of this hexaploid cell line to radiation and to Razoxane does not appear to differ greatly from that found for EMT6.

A hexaploid EMT6 tumour capable of growth *in vivo* has been derived from triploid cells taken from normal EMT6 tumours and suitably treated with Razoxane *in vitro*.

**ENHANCEMENT BY HYPERTHERMIA OF THE EFFECT OF BCNU ON THE EMT6 MOUSE TUMOUR.** P. R. TWENTYMAN, J. E. MORGAN and J. DONALDSON, *M.R.C. Clinical Oncology and Radiotherapeutics Unit, Hills Road, Cambridge.*

The combination of moderate hyperthermia (43°C water bath for 1 h) and 1,3 Bis (2-chloroethyl)-1-nitrosourea against the EMT6 mouse tumour has been studied. The effect was found to be much greater than would have been predicted from the effects of the single modalities, and this is true whether the end point be tumour cure, growth delay, or *in vitro* assay of clonogenic fraction. The results indicate that inhibition of "recovery from potentially lethal drug damage" is not the mechanism by which heat potentiates the action of BCNU.

**IDENTIFICATION OF PATHOGENIC FUNGI IN HUMAN MYCOSES BY THE USE OF LECTINS.** *R. W. STODDART and †B. M. HERBERTSON, **Strangeways Res. Lab. Cambridge. †Dept. Pathology, Tennis Court Road, Cambridge.*

Mycoses are among the most intractable infections in the immunodeficient patient. They are important and often fatal complications of some leukaemias and lymphomas and they may also arise in patients whose immune systems have been injured by cytotoxic drugs. The rapid identification of the infecting species is essential. This usually requires culture, especially where histological evidence is insufficient. Problems arise where culture is impossible or fails or where uncertainty remains as to the identity of the species grown with that seen in histological sections.

Differences in the carbohydrates of the cell walls of the various species can be exploited by use of fluorescein-labelled lectins, to circumvent these difficulties. Thus, *Candida albicans* stains strongly and selectively with concanavalin A for α-D-mannose, *Aspergillus fumigatus*, with soy-bean agglutinin for β-D-N-acetylgalactosamine and *Cryptococcus neoformans* with aprotinin probably for glucuronic acid. *Mucor*, *Blastomyces* and *Paracoccidiodes* can similarly be distinguished using these and other lectins specific for β-D-galactose, β-D-N-acetylglucosamine and α-L-fucose. The method is simple, rapid and practical.

**MELPHALAN AND STEROID HORMONES.** J. M. ANDERSON, T. ALGIE, J. K. GRANT and G. GETTINBY, *Royal Infirmary and Universities of Glasgow and Strathclyde, Glasgow.*

Striking differences in chemotherapy-related delay of recrudescence of mammary cancers in premenopausal and in postmenopausal women have been described in two recent trials (Fisher *et al.*, 1975, *New Engl. J. Med.*, **292**, 117; Bonadonna, Symposium of British Association of Surgical Oncology, Dec. 17, 1976). The longer cancer-free interval in premenopausal women may result from direct or pituitary-mediated suppression of ovarian functions. However, no significant differences in plasma oestradiol and androgens at both follicular and luteal phases have been observed in 6 node-positive patients menstruating regularly, during monthly 5-day pulses of oral melphalan. The differences between follicular and luteal levels of oestradiol were, as expected, statistically significant ($P < 0{\cdot}05$) in a Mann-Whitney U test, confirming the need to study putative chemotherapeutic effects at these phases separately.

Unexpectedly, plasma oestradiol levels were lower after similar pulses of melphalan in 6 node-positive postmenopausal patients ($P < 0{\cdot}05$ in Mann–Whitney U test) indicating that, although steroid hormones may be influenced by melphalan, an uncomplicated suppression of premenopausal ovaries is not the only factor worthy of investigation.

**MODULATION OF ALKYLATING AGENT TOXICITY AND ANTITUMOUR EFFECT BY STEROIDS.** R. Wilkinson and K. R. Harrap, *Inst. of Cancer Res., Sutton, Surrey.*

Prednisolone is frequently included in clinical treatment schedules with alkylating agents, where tumouricidal effects could be expected from both components. We have previously shown, however, that prednisolone in combination with chlorambucil potentiates cytotoxicity against cells in which predniso-lone alone has no effect (Harrap *et al.*, 1977, *Eur. J. Cancer*, **13**. More recent studies indicate that prednisolone may be combined with other alkylating agents with beneficial effects. By combining prednisolone with nitrogen mustard it is possible to suppress the toxicity of the alkylating agent, whilst enhancing its antitumour effect. In the case of binary combinations of melphalan and prednisolone, both toxicity and antitumour effect are increased above those of the alkylating agent alone. These effects are closely dependent upon schedule design and, by administering the steroid after the alkylating agent, toxicity can be reduced with no loss of antitumour effect. In sensitive cells, alkylating agent cytotoxicity is accompanied by morphological dissociation of chromatin and changes in nuclear protein integrity, which are not detected in resistant cells. However, binary combinations of alkylating agents and steroids are cytotoxic in resistant cells, and elicit a biochemical response typical of the sensitive cells. It is proposed that these experimental observations may provide a more rational basis for the design of clinical treatment schedules.

**THE MODE OF ACTION AND BIOLOGICAL EFFECTS OF CERTAIN ANTITUMOUR DRUGS AND ANTIFERTILITY AGENTS IN *XENOPUS LAEVIS* AND *MUS MUSCULUS*.** B. N. Hemsworth and A. A. Wardhaugh, *Life Science Laboratory, Teesside Polytechnic, Middlesbrough.*

Using an *in vitro* toad system, described by Jones and Jackson (1974, *J. Reprod. Fertil.*, **38**, 347) for the detection and study of chemical mutagens able to affect post-meiotic germ cells, we have found that exposure of sperm to the isomeric forms of dimethylmyleran, MMS, gamma radiation and nicotine leads to embryopathy. In high concentration, various steroids have an antifertility action due to impaired sperm motility. Studies indicate that lower concentrations are compatible with fertility and production of apparently normal offspring.

In *Mus musculus*, the isomeric forms of dimethylmeran have an antifertility action referable to interference with spermatogonia, spermatocytes and post-meiotic germ cells.

**CHEMOTHERAPY OF PIG LYMPHOMA.** S. E. Brownlie, J. G. Campbell, K. W. Head, P. Imlah, and H. S. McTaggart, *Faculty of Veterinary Medicine, University of Edinburgh*, and J. G. McVie, *Department of Clinical Oncology, University of Glasgow.*

A preliminary report at the BACR meeting in March 1977 described experiments to investigate the applications of pigs with hereditary lymphoma (Head *et al.*, 1974, *Vet. Rec.* **95**, 523) as a chemotherapy model for man. Cases have been given single-agent therapy, after toxicity tests. Prednisolone intramuscularly (4 cases) and orally (2 cases) for 5 weeks, was shown to improve vigour and appetite, red-cell picture, neutrophil and platelet counts. Circulating tumour-cell counts and lymph-node size were reduced to normal. Relapse occurred 5 weeks after withdrawal of the drug. One 18-month-old pig was treated for 2 months with prednisolone and improved dramatically, but after another 2 months became recumbent and refused to eat. Autopsy showed a lymphomatous mass in the meninges pressing on the brain. Tumour has not been seen at this site in any of 50 untreated cases, and it provides a good analogy to lymphocytic leukaemia in children. An almost moribund 4-month-old female improved on treatment with prednisolone, followed by vincristine and cyclophosphamide. The last two drugs given 48 h apart showed better results than when given simultaneously. Remission was maintained for over a year and the pig looked normal. It showed regular oestrus, which is not seen in untreated cases. Side effects from cyclophosphamide are almost identical to those in man, particularly a consistent reversible neutropenia at Days 6–8. Vincristine caused hindleg ataxia in the pig and doxorubicin produced liver failure, alopecia, nail changes and cardiotoxicity. The therapeutic response and comparable side effects to cytotoxic drugs suggest that the pig lymphoma is a useful model for testing new agents and combinations.

### COMBINATION CHEMOTHERAPY IN MALIGNANT MELANOMA.

M. Soukop, K. C. Calman, J. G. McVie and R. MacKie, *Department of Clinical Oncology, Glasgow.*

The aim of this study was to compare two regimes of combination chemotherapy in disseminated malignant melonoma. All patients had biopsy-proven disease, and sites of involvement included skin, lymph nodes, liver, brain and lung. Response was defined by the standard criteria used by the Eastern Cooperative Oncology Group.

The first regime consisted for DTIC (200 mg/m$^2$ i.v. daily for 5 days) together with methyl CCNU (100 mg/m$^2$ orally on Day 1) and vincristine (1 mg/m$^2$ i.v. on Day 1) the regime being repeated at 3 or 4-weekly intervals. Toxicity was mainly related to nausea and vomiting. Fourteen patients were treated and all were evaluable. There was one partial response.

The second regime was chosen fortuitously and, because a response was obtained, it was evaluated in a series of patients. This drug regime consisted of adriamycin (50 mg/m$^2$ i.v. Day 1) cyclophosphamide (500 mg/m$^2$ i.v. Day 1), vincristine (1 mg/m$^2$ i.v. Day 1) and prednisolone (50 mg orally daily Days 1–5. Ten patients were evaluable and in this group there were 6 partial responses.

Although adriamycin is not reported to be an effective single agent, the above combination certainly produced a higher response rate than that reported from conventional chemotherapy in advanced malignant melanoma. Further work will be aimed at defining the active drugs in the combination. In conclusion the combination of adriamycin, cyclophosphamide, vincristine and prednisolone produced 6/10 partial responses compared to 1/14 using conventional therapy with DTIC, methyl CCNU and vincristine.

### RAZOXANE/ARA-C IN THE TREATMENT OF ADULT ACUTE LEUKAEMIA.

K. Hellmann, K. A. Newton and J. G. Humble, *Departments of Oncology and Haematology, Westminster Hospital, London.*

Razoxane has shown actively in acute lymphoblastic and in acute myeloblastic leukaemia (AML) of children (Hellmann *et al.*, 1969, *Brit. med. J.*, *i*, 822; Krepler and Pawlowsky, 1975, *Osterreichische Z. Onkol.*, **2**, 112). Since AML has a particularly poor prognosis in the elderly, and because treatment of this group of patients presents considerable problems, razoxane was combined with cytosine arabinoside (the mainstay of AML treatment). The regime consisted of razoxane 125 mg 3× daily for 3 days and Ara-C 1 mg/kg i.v. on the same 3 days. The course was repeated every 5–7 days. Toxicity, if any, was chiefly haematological, but when normal blood elements declined, they began to recuperate after a few days. Of 5 patients with acute monocytic or myelomonocytic leukaemia, 1 did not respond, 3 had partial remissions and 1 had a complete remission. The regime is suitable for outpatient administration and no special nursing care is required. Supportive care required when patients were in hospital was minimal. A striking feature of the response in 3 of the patients was the rapidity of onset of the blast clearance of the blood. From what is known of the clinical activity of the two drugs it would be most unlikely that the results so far could have been obtained with either of the drugs alone, but whether they potentiate each other or are merely good additive agents is not known as yet.

### CHEMOTHERAPY FOR LOCALIZED TESTICULAR TERATOMAS.

K. Höffken and C. G. Schmidt, *Cancer Research Campaign Laboratories, University of Nottingham and Dept. of Internal Medicine (Cancer Research), University of Essen, West Germany.*

26 patients with Stage II testicular teratomas (metastases confined to retroperitoneal lymph nodes (RLN)) were treated by cytostatic chemotherapy with or without radiotherapy after retroperitoneal lymph node dissection (RLD). Regimens consisted of both single and combination chemotherapy. In a retrospective analysis after a mean follow-up time of 35 months (range 27–60 months) 42% relapsed with distant metastases. Although not statistically significant ($\chi^2 = 0{\cdot}98$; $P = 0{\cdot}30$) 56% of Stage IIB patients (considerable metastates to RLN; partial RLD performed) showed dissemination, compared with only 35% of patients with Stage IIA disease (minimal metastases to RLN; total RLD performed). Median relapse-free survival is not yet reached (57% relapse-free survival after 5 years). Median relapse-free interval was 15 months, compared with 3 months, in a retrospective analysis of patients being referred with widespread disease after watch-policy only. Median total survival is more than 60 months as evaluated by life-table analysis (5-year survival rate 71%). No difference was found in patients given additional radiotherapy. These results indicate a more favourable outcome for patients being treated by adjuvant chemotherapy than the historical prognosis (5-year survival rate 40–50%). Prospective studies have to confirm these findings, since historical data embody many variables, thus not accurately reflecting differences between untreated and cytostatically treated patients with Stage II testicular teratomas.